# Abnormality of anxious behaviors and functional connectivity between the amygdala and the frontal lobe in maternally deprived monkeys

**DOI:** 10.1002/brb3.3027

**Published:** 2023-07-18

**Authors:** Xiao‐Li Feng, Jiao‐Jian Wang, Jing Wu, Xiao‐Feng Ren, Hui Zhou, Si‐Yu Li, Jie Zhang, Sheng‐Hai Wang, Yun Wang, Zheng‐Fei Hu, Xin‐Tian Hu, Tian‐Zi Jiang

**Affiliations:** ^1^ Key Laboratory of Animal Models and Human Disease Mechanisms of the Chinese Academy of Sciences & Yunnan Province, Kunming Institute of Zoology Chinese Academy of Sciences Kunming Yunnan China; ^2^ Department of Physiology Faculty of Basic Medical Science Kunming Medical University Kunming Yunnan China; ^3^ Institute of Neuroscience Kunming Medical University Kunming Yunnan China; ^4^ State Key Laboratory of Primate Biomedical Research Institute of Primate Translational Medicine Kunming University of Science and Technology Kunming China; ^5^ Yunnan Key Laboratory of Primate Biomedical Research Kunming China; ^6^ Kunming College of Life Science University of Chinese Academy of Sciences Kunming Yunnan China; ^7^ National Resource Center for Non‐Human Primates, Kunming Primate Research Center, and National Research Facility for Phenotypic & Genetic Analysis of Model Animals (Primate Facility), Kunming Institute of Zoology Chinese Academy of Sciences Kunming Yunnan China; ^8^ Center for Excellence in Brain Science Chinese Academy of Sciences Shanghai China; ^9^ Brainnetome Center and National Laboratory of Pattern Recognition Institute of Automation Chinese Academy of Sciences Beijing China; ^10^ Research Center for Augmented Intelligence Zhejiang Laboratory Hangzhou China; ^11^ Center for Excellence in Brain Science Institute of Automation Chinese Academy of Sciences Beijing China; ^12^ School of Basic Medical Sciences Kunming Medical University Kunming Yunnan China

**Keywords:** amygdala–prefrontal connection, anxious behaviors, brain circuits, maternal deprivation, rhesus monkeys

## Abstract

**Objective:**

Anxious behaviors often occur in individuals who have experienced early adversity. Anxious behaviors can bring many hazards, such as social withdrawal, eating disorders, negative self‐efficacy, self‐injurious thoughts and behaviors, anxiety disorders, and even depression. Abnormal behavior are is closely related to changes in corresponding circuit functions in the brain. This study investigated the relationship between brain circuits and anxious behaviors in maternal‐deprived rhesus monkey animal model, which mimic early adversity in human.

**Methods:**

Twenty‐five rhesus monkeys (*Macaca mulatta*) were grouped by two different rearing conditions: 11 normal control and mother‐reared (MR) monkeys and 14 maternally deprived and peer‐reared (MD) monkeys. After obtaining images of the brain areas with significant differences in maternal separation and normal control macaque function, the relationship between functional junction intensity and stereotypical behaviors was determined by correlation analysis.

**Results:**

The correlation analysis revealed that stereotypical behaviors were negatively correlated with the coupling between the left lateral amygdala subregion and the left inferior frontal gyrus in both MD and MR macaques.

**Conclusion:**

This study suggests that early adversity‐induced anxious behaviors are associated with changes in the strength of the amygdala–prefrontal connection. The normalization of the regions involved in the functional connection might reverse the behavioral abnormality. It provides a solid foundation for effective intervention in human early adversity.

**Significance Statement:**

This study suggests that early adversity‐induced anxious behaviors are associated with changes in the strength of the amygdala–prefrontal connection. The higher the amygdala–prefrontal connection strength, the less stereotyped behaviors exhibited by monkeys experiencing early adversity. Thus, in the future, changing the strength of the amygdala–prefrontal connection may reverse the behavioral abnormalities of individuals who experience early adversity. This study provides a solid foundation for effective intervention in humans' early adversity.

## Graphical abstract

Up to now, few studies have investigated the relationship between brain circuits and anxious behaviors in maternally deprived rhesus monkeys. The functional image data were obtained in order to detect any changes in functional connectivity between emotion‐related brain regions in maternally deprived monkeys, and the anxious behaviors were recorded to investigate the behavioral changes. We subsequently further examined the relationship between the correlation between behavioral changes and the functional connectivity was further studied. The duration of stereotyped behaviors in maternally deprived (MD monkeys) and peer‐reared monkeys (PR monkeys) was significantly longer than the mother‐reared monkeys (MR monkeys), with significant differences in functional connectivity between the left lateral amygdala (basolateral region) and left prefrontal gyrus. Besides, the relationship between the functional connectivity between the amygdala and the frontal lobe and the abnormal changes in anxious behaviors in MD monkeys was also established. This study might provide solid basis for effective intervention in human early adversity.

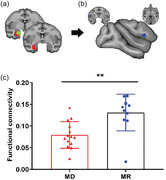

## 1 INTRODUCTION

In humans, anxious behaviors often occur in individuals who have experienced early adversity (McCrory et al., 2012). Anxious behaviors can bring many hazards, such as social withdrawal, eating disorders, negative self‐efficacy, self‐injurious thoughts and behaviors, anxiety disorders, and even depression (Ge et al., 2021; Lapp & Croy, 2021). Abnormal behaviors are closely related to changes in corresponding circuit functions in the brain (Engels et al., 2010; C. E. Han et al., 2013). Numerous studies have demonstrated that the amygdala, located deep in the temporal lobe of the brain, act as a central regulator of negative emotions. The two subregions of the amygdala, namely the central amygdala nucleus and basal amygdala nucleus, are closely associated with anxious behaviors (Ancelin et al., 2021; Kooiker et al., 2021). Moreover, the prefrontal cortex is also known to play a key role in the regulation of anxious behaviors (Hart & Rubia, 2012; McCrory et al., 2010). The relationship between these two brain regions may be directly related to anxious behavior.

In humans, after individuals show persistent anxiety symptoms, retrospective studies are difficult to quantitatively control other potential influencing factors. Hence, it is crucial to elucidate the relationship between brain connection circuits of the amygdala and prefrontal cortex and anxious behaviors in taking the anl model of early adversity as the research object.

In rats, adolescent males have been reported that those that have experienced early adversity and stress showed abnormal functional connections in the reward and fear pathways in the brain and increased structural connections between the amygdala and the medial prefrontal cortex (Bolton et al., 2018). As to non‐human primate models, to date, few studies have used non‐human primate model for early adversity to explore the relationship between brain circuits and anxious behaviors, especially at a young age.

Non‐human primate models are vital to understanding the mechanisms behind the development and expression of human psychopathology (Kalin & Shelton, 2003; Nelson & Winslow, 2009). Indeed, in a previous study, our team indicated that the negative impact of postnatal maternal deprivation on rhesus monkeys cannot be reversed by long‐term normal social life (1.5 years; Feng et al., 2011). However, many studies in rodents have shown that the negative effects of maternal deprivation can be reversed by enriching the environment (Cordier et al., 2021; Odeon & Acosta, 2019). The contradictory results showed that the negative effects of maternal deprivation in rhesus monkeys are lasting, which were very similar to the long‐term effects of early adversity in humans. Moreover, the most common and stable negative behaviors observed in infant monkeys separated from mothers are anxious behaviors. Therefore, compared with rodent models, it is more appropriate to take maternally deprived monkeys as an early stress animal model to study the relationship between brain connection circuits of the amygdala–prefrontal cortex and anxiety behavior.

Therefore, this study plans to study the brain circuit mechanism of anxiety behaviors in rhesus monkeys that strictly controls the length and intensity of maternal deprivation. In addition to its biological significance, the study also has important social implications. If we can obtain clear research results on the brain circuit mechanism of anxiety behavior, then it will hold high theoretical guiding value for the early cultivation of children who experience early adversity. It is hypothesized that MD monkeys have different amygdala–prefrontal cortex connectivity and anxiety behavior, compared to the mother‐reered monkeys.

## 2 MATERIALS AND METHODS

### 2.1 Subjects

Twenty‐five rhesus monkeys (*Macaca mulatta*) were grouped by two different rearing conditions: normal control and mother‐reared monkeys (MR group, *n* = 11) and maternally deprived and peer‐reared monkeys (MD group, *n* = 14), as shown in Table 1. The MR infants lived with mother monkeys in isolated social groups. Each group consisted of one adult male and four or five adult females and their babies. Each social group lived in an interconnected internal community (2.60 × 2.50 × 2.60 m) outdoors (2.70 × 2.70 × 2.70 m). Monkeys could drink running water freely all day. In addition, they were provided with fruits and vegetables once per day. For the MR monkeys, when they reached 6 months of age, each infant monkey was weaned. They were then transferred to a steel cage (0.75 × 0.70 × 0.75 m) corresponding to their age.

**TABLE 1:** The basic information of the 25 monkeys (MD group, *n* = 14; MR group, *n* = 11).

**Monkey number**	**Gender**	**Group**	**Age**
09003	Male	MD	4
09007	Male	MD	4
09031	Male	MD	3
10004	Female	MD	3
10006	Female	MD	3
10017	Male	MD	3
10019	Male	MD	3
10402	Female	MD	3
10441	Male	MD	2
11008	Female	MD	2
11010	Female	MD	2
11022	Female	MD	2
11085	Male	MD	2
11368	Female	MD	2
09317	Male	MR	4
09321	Male	MR	4
09381	Male	MR	4
10046	Female	MR	3
10405	Male	MR	3
11017	Male	MR	2
11028	Female	MR	2
11048	Female	MR	2
11053	Male	MR	2
11094	Female	MR	2
11321	Male	MR	2

Abbreviations: MD group, maternally deprived and peer‐reared monkeys; MR group, normal control and mother‐reared monkey

The MD monkeys separated from their mothers at birth are as follows: (1) approximately 60% of MD babies were newborns and separated from their caregivers due to their mothers’ inexperience. Inexperienced mothers might mistreat their first child, leading to serious injury and death. Therefore, in this case, the keeper of the primate center must take the baby into an incubator to be bred; (2) nearly 20% of the MD infant monkeys were separated from their mothers due to lack of breast milk of the mother monkey; (3) approximately 20% of MD monkeys were born on rainy or cold days, which can lead to illness or death in newborn monkeys. To prevent this, monkey keepers sometimes took newborn monkeys away from their mothers when they got wet and keep them in an indoor incubator until the weather improves. However, mothers often refused to take the monkeys back, and the monkeys might continue to be kept in incubators.

MD monkeys were raised in pairs for the first month of life, kept in an incubator at 32°C, and swaddled with clean towels. During this month, the baby monkeys were fed 8–9 times a day. The infant pair was then transferred to a stainless steel cage (0.75 × 0.70 × 0.75 m). At 6–7 months of age, MR monkeys and MD monkeys were raised in an indoor (2.60 × 2.50 × 2.60 m) to outdoor (2.70 × 2.70 × 2.70 m) colony.

### 2.2 Behavioral data collection and analysis

The tripod was fixed to the ground and secured with a digital camera approximately 5 m from the cage (to avoid disturbing the monkeys). Each monkey was examined for 15‐min recordings four times a week, giving each monkey a total of 2 h (eight videos). Later, behavioral analysis was performed on a computer.

Each film focused on a different monkey. The anxious behaviors in monkeys were analyzed as follows. When the monkey started to act nervously (including mainly stereotyped behaviors, defined below) in the video, the analyzer paused the video to record the exact moment. Then, the anxious behavior was recorded until the anxious behavior stopped. The time (in seconds) between these two time points was considered a situational event for anxious behavior. Fifteen minutes of video could contain many episodes. The duration of the anxious behaviors was calculated by adding up all the episodes of anxious behavior. Then, this duration of the monkey's anxious behaviors was divided by the total time of the monkeys’ total recordings (minutes, 8 × 30 = 240 min) to obtain a unit value of s/min, which was used for subsequent analysis.

### 2.3 Behavioral categories and definitions

Stereotyped behaviors are the mainfestation of anxious behaviors, typically characterized by repetitive, invariant patterns behavior that lack an obvious goal or function. Macaques can exhibit a wide range of stereotyped behaviors, including pacing, swing, bouncing, self‐biting, self‐grasping, and so on (Hwang et al., 2020; also see Bessa Ferreira et al., 2018; Ramsey et al., 2018). Pacing is a repetitive pattern of activity that follows an invariant path usually involving circling the cage. Swing is the movement of the upper body back and forth without moving the feet. Bouncing is a repeated jump up and down. Flipping is grabbing the top of the cage and flipping the body. Shaking of the cage includes any violent shaking of the cage. Self‐grasping means grabbing a part of the monkey's own body. Self‐biting includes any bite marks on the monkey's body. Sucking involves sucking on fingers or toes. Hair extraction is the use of fingers or teeth to pluck out the monkey's hair. Clapping hands mean picking up them lips and moving them to make a smacking sound. Body spasming is rapid tremors of the body. Stereotyped behaviors were quantified by duration. The technicians who analyzed the video were blind to the groups of monkeys. Comparisons of duration of stereotyped behaviors between MD and MR monkeys were performed using the Mann–Whitney U‐test (for data not satisfying normal distribution) or independent samples *t*‐test (for data satisfying normal distribution). Data were shown as mean ± standard error of the mean, with significance at *p < .05, **p < .01, ***p < .001, and ****p < .0001. All *p*‐values were generated by two‐tail tests.

### 2.4 Magnetic resonance imaging (MRI) data analysis

#### 2.4.1 MRI data acquisition

The MRI data for all rhesus monkeys were obtained using Siemens MAGNETOM Verio 3.0 T (Siemens 3.0 Tesla MRI Scanner). Before the scan, each animal was anesthetized with intravenous propofol (12 mg/kg) and atropine (0.05 mg/kg). Propofol was administered intravenously at a rate of 12 mg/kg/min. The acquisition parameters for the diffusion tensor imaging (DTI) data were as follows: field of view (FOV) = 131 ×131 mm, acquisition matrix = 84 × 84. Echo time (TE) = 92, repeat time (TR) = 9000 MS, flip angle = 90 degrees, 38 slices, slice thickness = 1.6 mm and no gap. The DTI data include 64 images with diffusion gradients (***b*** = 1000 s/mm^2^) and one non‐diffusion‐weighted image (***b*** = 0 s/mm^2^). The following parameters were used to collect functional magnetic resonace imaging (fMRI )data: TR = 2000 ms; TE = 30 ms; 25 slices; planar resolution of 2.40 × 2.40 mm^2^; cross‐section thickness of 2.4 mm; and 180 volumes. The sagittal 3D structural T1 images were acquired for registration. The scan parameters were: TR = 2000 ms, TE = 2.98 ms, FOV = 128 × 128 mm, acquisition matrix = 128 × 128, slice thickness = 1 mm.

#### 2.4.2 Resting‐state fMRI data preprocessing

Rs‐fMRI data were analyzed with FSL and SPM8 software. The first 10 volumes in each feature set were removed. The slice time of the remaining images was corrected, and the images were rearranged to the first volume to correct for head movement. All fMRI images were registered in structure images of skull dissection and further normalized to rhesus monkey INIA19 template using the FSL software. Then, the normalized fMRI data were resampled into 1 mm voxel and were smoothed using a full‐width 3 mm half‐maximum(FWHM) Gaussian kernel (. Next, head motion, white matter, cerebrospinal fluid, and mean global signals were regressed out. Finally, the functional images were filtered with a temporal band path of 0.01–0.1 Hz.

#### 2.4.3 Definition of amygdala masks

The amygdala is closely linked to the cerebral cortex and subcortical regions and is a key hub for dealing with threats and coordinating complex emotional and physiological responses (H. J. Han et al., 2014). Since the traditional partitioning of the amygdala in monkeys is too small for MRI analysis, we use our own partitioning results for further analyses. To define amygdala subregions, the bilateral entire amygdala was first extracted from the INIA19 rhesus template. Then, the structural T1 image of each monkey was transformed into the INIA19 template, and an inverse transformation was performed to transform the bilateral amygdala masks into structural T1 space. Next, T1 image was co‐registered to the same subject's non‐diffusion‐weighted image (b = 0 s/mm^2^), and a transformation matrix was finally applied to transform bilateral amygdala masks in the structural T1 space to the diffusion space for fiber tracking.

#### 2.4.4 DTI data preprocessing and fiber tracking

Distortions in the diffusion‐weighted images caused by eddy currents and simple head motions were corrected using FSL software. After the amygdala mask transformed to diffusion space, the fiber tracking of each voxel of amygdala was performed to obtain its whole brain anatomical connectivity patterns. Fiber tracking was performed using the FSL package, and voxel‐wise estimates of the fiber orientation distribution were calculated. The probability distributions were computed for two fiber directions at each voxel (Behrens et al., 2007). Drawing on these distributions, we estimated the fiber tracts between each voxel in the amygdala and every voxel of the whole brain. This approach drew a sample from each fiber orientation distribution at the current voxel and chose the sample closest to the orientation identified in the previous step. The connection probability between each voxel in the seed region and any other voxel in the brain was the number of traces arriving at the target site. To facilitate data storage, all the connectivity profiles for each voxel were down‐sampled to 3 mm isotropic voxels (Johansen‐Berg et al., 2004; Wang et al., 2012). Cross‐correlations (dimensions: number of seeds × number of seeds) between the connectivity patterns of all voxels in the seed mask were calculated and used for automatic parcellation. The (seed*i, seedj*) element value of the cross‐correlation was defined by the correlation between the connectivity profile of seed *i* and the connectivity profile of seed *j*.

#### 2.4.5 Tractography‐based parcellation

The cross‐correlation matrix then underwent spectral clustering segmentation by automated clustering to define different clusters (Wang et al., 2012). The goal of clustering the cross‐correlation matrix was to group together regions that shared similar connectivity with other voxels of the brain. In the end, each of these seed regions was parcellated into the maximum number of areas that had the greatest continuity or allowed the smoothest parcellation result.

To take into account inter‐individual differences in the parcellation results, we calculated the maximum probability map (MPM) to show the final parcellation results of the macaque amygdala. The advantage of an MPM is that it reflects the volumes and locations of the subregions obtained by a tractography‐based parcellation more precisely than other methods, which are based on simply thresholding the original probability maps (Eickhoff et al., 2006). To do this, we first transformed the individual parcellation results from diffusion space to the INIA19 template. The population‐based MPM was calculated based on all 24 transformed macaque parcellation results. The MPM was calculated by assigning each voxel to the cluster in which it was most likely to be located. If two clusters showed the same probability at a particular voxel, this voxel was assigned to the cluster that had the higher average probability among the immediately adjacent voxels (Wang et al., 2012).

In order to determine the number of subregions of the amygdala, we used silhouette value and overlap degree defined using Dice coefficient to identify the optimal cluster numbers.

#### 2.4.6 Resting‐state functional connectivity (RSFC) analyses of amygdala subregions

After obtaining the amygdala subregions, the whole brain RSFC characterized using the Pearson correlation coefficient of each amygdala subregion was calculated. We first sample the left and right amygdala subregions’ masks into 1 mm cubic. Then, we obtained the mean time series and computed the whole brain resting‐state functional connections of each amygdala subregion. Next, in order to improve the normality, the correlation coefficient *r*‐values were converted to *z*‐values using Fisher's *z* transformation. Finally, two‐sample *t*‐test in a voxel‐wise manner was used to identify the exact regions that differed in their RSFC strengths of each amygdala subregion between maternal separation macaques and normal control macaques. The significance was determined with a cluster‐level corrected threshold of *p* < .05 (cluster‐forming threshold at voxel‐level *p* < .01 and cluster size 24 mm^3^ using the AlphaSim).

#### 2.4.7 Correlation analyses of RSFC and behavioral performances

After obtaining the brain areas with significant functional differences in maternal separation and normal control macaques, the correlation analyses were performed to determine the relationship between functional connectivity strengths and behavioral measurements of macaque with significance at *p < .05, **p < .01, ***p < .001, and ****p < .0001.

## 3 STATISTICS

In Figure 2, we used Mann–Whitney U‐test (the data do not follow a normal distribution) and Figure 4 we used independent samples *t*‐test (the data follow a normal distribution), respectively. For the correlation between stereotyped behaviors and the connectivity between the left lateral amygdala subregion and left IFG, we used Spearman correlation analysis (Figure 5).

## 4 RESULTS

**FIGURE 1:**
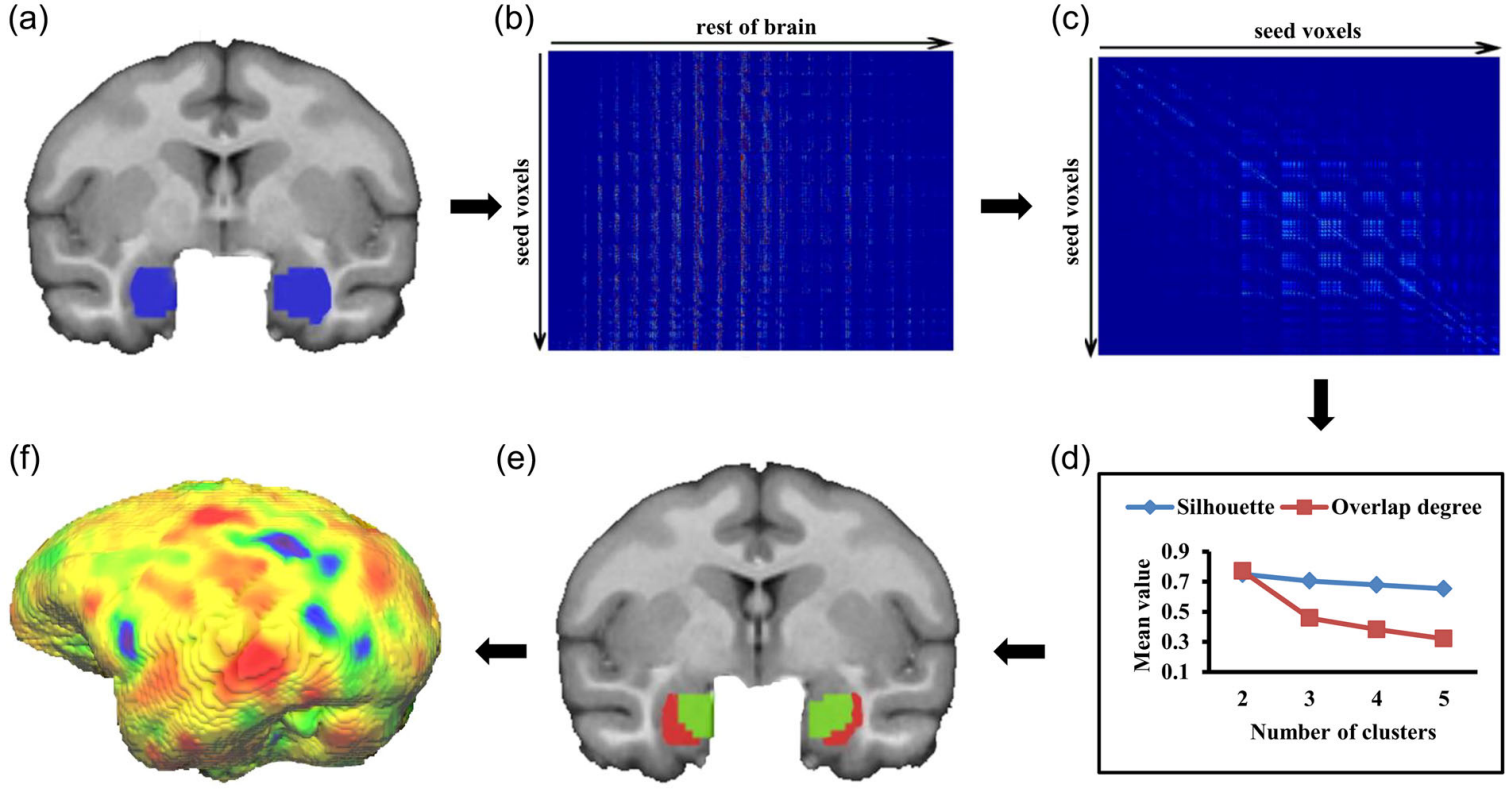
The framework of this study. (A) Definition of the bilateral amygdala in rhesus monkeys, (B) mapping the whole brain anatomical connectivity pattern for each voxel in amygdala, (C) calculating the whole brain anatomical connectivity similarity of each pair of voxels in amygdala (D) selecting the number of clusters based on silhouette and overlap degree indices, (E) parcellation results of amygdala, (F) whole brain functional connectivity analysis for each amygdala subregion.

The framework of this study is shown in Figure 1.

### 4.1 The stereotyped behaviors

**FIGURE 2:**
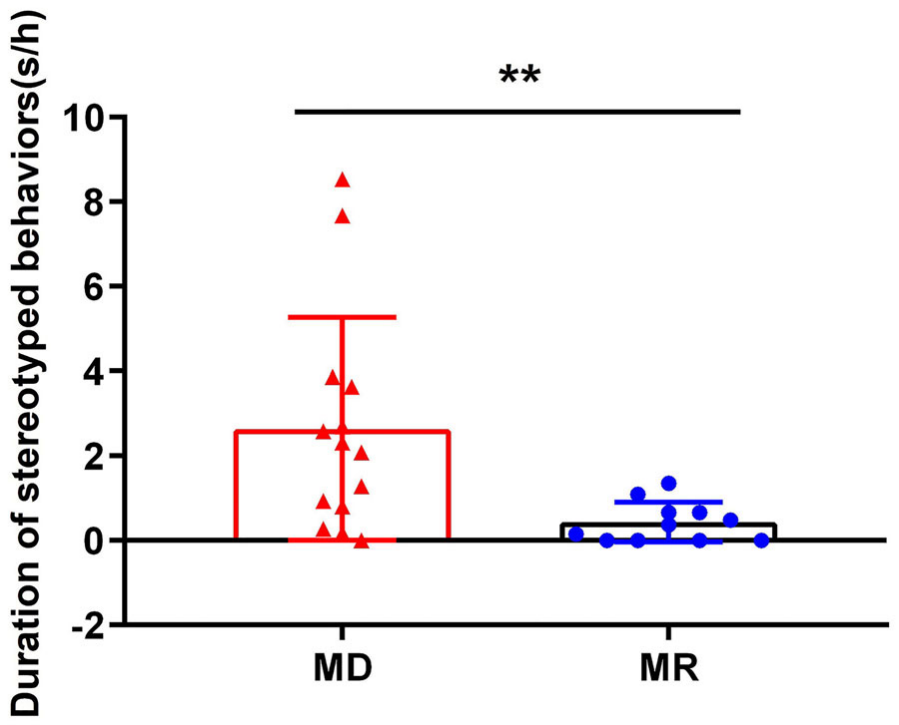
Comparison of the duration of stereotyped behaviors between the maternally deprived and peer‐reared (MD) monkeys and normal control and mother‐reared monkey (MR) monkeys. Compared to MR monkeys (*n* = 11, blue dots), the duration of stereotyped behaviors in MD monkeys (*n* = 14, red triangles) was significantly increased (Mann–Whitney U‐test, *U* = 26, *p* = .0039).

The duration of stereotyped behaviors in MD monkeys was significantly longer than in the MR monkeys (*p* = .0039; Figure 2). Considering that there were both males and females in the MD (*n* of females = 7, *n* of males = 7) and MR (*n* of females = 4, *n* of males = 7) monkeys, we further compared the duration of stereotyped behaviors between male and female monkeys within the MD and MR group, respectively. The results showed that there was no significant difference in the duration of stereotyped behaviors between females and males in either MD (independent samples *t*‐test, *t* = 1.331, *p* = .2080) or MR monkeys (Mann–Whitney U‐test, *U* = 11, *p* = .5606).

### 4.2 Anatomical connectivity‐based parcellation of the amygdala

**FIGURE 3:**
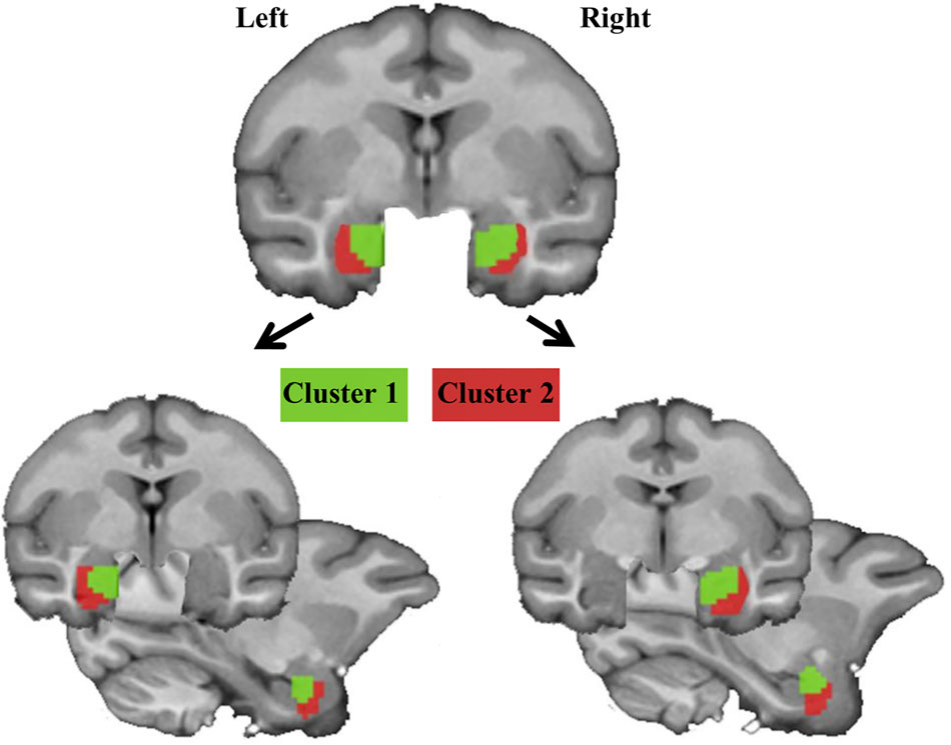
Anatomical connectivity based parcellation of bilateral amygdala. Anatomical connectivity‐based parcellation identified two subregions in left and right amygdala. One cluster is located in the lateral amygdala (red one) and the other one is located in the medial amygdala (green one).

According to the anatomical position structure, the division identifies the subregions of the two hemispheres of the left amygdala and the right amygdala (Figure 3). The median area (medial cortical region) is green (cluster 1) and the lateral area (basolateral region) is red (cluster 2). This is the method to divide the left and right amygdala subregions, and the two regions are selected for static functional connectivity analysis with the inferior frontal gyrus (IFG).

### 4.3 RSFC analyses

**FIGURE 4:**
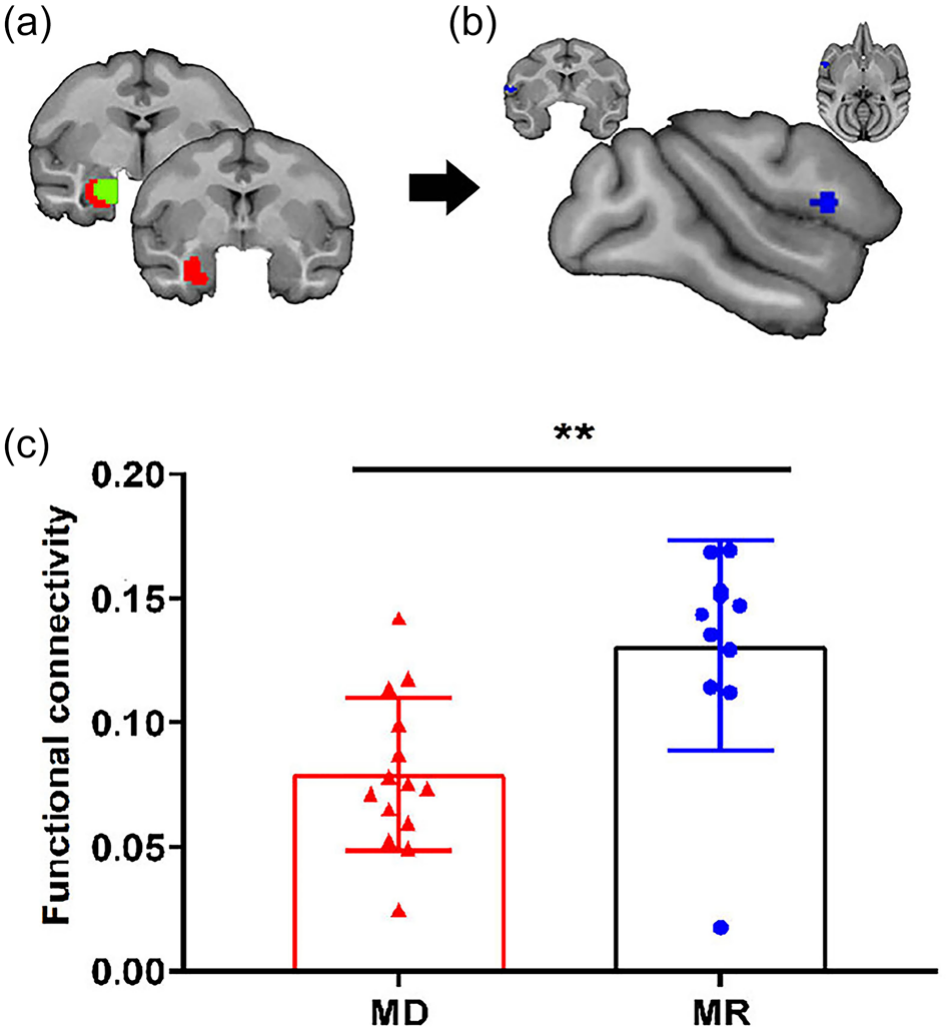
Whole brain functional connectivity differences of amygdala subregion in MD monkeys. Whole brain functional connectivity analysis identified significantly lower functional connectivity between left lateral subregion of amygdala and the left inferior frontal gyrus (IFG) in MD than MR monkeys. After calculating the mean FC between left lateral amygdala subregion and IFG, compared to MR monkeys (*n* = 11, blue dots), the functional connectivity in MD monkeys (*n* = 14, red triangles) was significantly decreased (independent samples *t*‐test, *t* = 3.552, *p* = .0017).

Whole brain RSFC analysis identified significant differences in functional connectivity between the left lateral amygdala (basolateral region) and left IFG in MD monkeys (27 voxels, peak *t* = −3.88, Figure 4a, b, c). The mean functional connectivity between the left lateral amygdala subregion and the left IFG was calculated in MR and MD groups and also showed reduced functional connectivity（FC） in MD monkeys than that in MR monkeys (*p* = .0017; Figure 4c). Similarly, we performed the comparison of functional connectivity between males and females within the MD and MR monkeys, respectively. The results showed that there was no significant difference in functional connectivity between females and males in either MD (independent samples *t*‐test, *t* = 0.9267, p = .3724) or MR monkeys (Mann–Whitney U‐test, *U* = 4, *p* = .0727).

**FIGURE 5:**
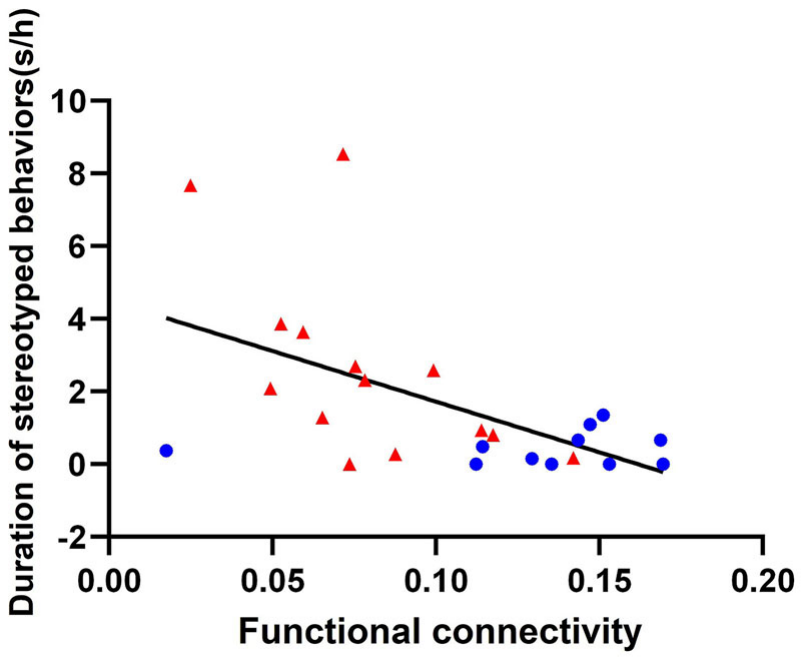
The correlation between stereotyped behaviors and the connectivity between left lateral amygdala subregion and left IFG in both MD and MR monkeys. Stereotyped behaviors were negatively correlated with the coupling between the left lateral amygdala subregion and the left IFG in both MD (*n* = 14, red triangles) and MR (*n* = 11, blue dots) monkeys (Pearson correlation analysis, *r* = −0.5451, *p* = .0048).

### 4.4 Correlation of behaviors and functional connection

Correlation analysis was performed to establish the changed functional connectivity and behavior. The correlation analysis revealed that stereotyped behaviors were negatively correlated with the coupling between the left lateral amygdala subregion and the left IFG in both MD and MR monkeys (*r* = −0.5451, *p* = .0048). That is, the individuals who had higher functional connection strength between the left lateral amygdala and IFG exhibited less stereotyped behaviors, and they might be less anxious than those with less coupling intensity.

## 5 DISCUSSION

In this study, we directly linked behavioral abnormalities to changes in brain function in monkeys. Moreover, previous human studies usually adopted retrospective interviews to index early adversity, which might not be strictly controlled. Conversely, early adversity (maternal deprivation) was controlled strictly and quantitatively in the present study, and definite results were obtained.

Under strictly controlled maternal deprivation, the stereotyped behaviors of maternal deprivation monkeys increased, compared with normal control monkeys, which indicated that the intrinsic state of the maternally deprived monkeys (the MD group) was much more anxious than that of the mother‐reared monkeys (the MR group).

On the one hand, the coupling values of the left lateral amygdala (basolateral region) and left IFG in both the MD and MR groups were positive, and the averaged coupling strength of the MR group was significantly enhanced, which might indicate that the functional coupling of the left amygdala and left lateral IFG might be more stable in the MR group.

On the other hand, stereotyped behaviors were negatively correlated with the coupling strength between the left lateral amygdala (basolateral region) and left IFG in both the MD and MR groups. That is, the individuals who had higher connection intensity between the left lateral amygdala and IFG exhibited less stereotyped behaviors, and they might be less anxious than those with less coupling intensity.

These results collectively suggested that postpartum maternal deprivation not only leads to abnormalities in both behaviors and brain connections but also affects the correlations between these two facets. That is, there is a strong link between abnormal behavior and abnormal changes in brain connections. It is well known that the amygdala is a key structure for emotional regulation (Gabard‐Durnam et al., 2014; Hulvershorn et al., 2014; Kim et al., 2011). The IFG also plays an important role in emotional responses (van Harmelen et al., 2014; Yurgelun‐Todd, 2007). In this study, the connection intensity of these two regions was inversely correlated with the duration of stereotyped behaviors, which reflected the emotional state of inner anxiety. This result demonstrates the importance of the amygdala and the IFG in normal emotional development. More specifically, the neural circuit composed of these two brain regions played an important role in mediating intrinsic anxiety states. The increased connection intensity was significantly related to the decreased anxious behavior. In the MD group, anxious behaviors were significantly increased, and the coupling stringency of the left lateral amygdala (basolateral region) and IFG was weaker than that of the MR group.

The amygdala is responsible for low‐level emotional regulation (Boehme et al., 2015; Laeger et al., 2012) and thus plays a large role in the instinctive emotional response (Girotti et al., 2018; Quirk & Beer, 2006). In contrast, as a region that formed much later in the process of evolution (Yurgelun‐Todd, 2007), the IFG deals with a good number of higher brain functions, such as the integration of emotional information and involvement in emotion‐related cognition (Girotti et al., 2018; Jasinska et al., 2012; van Harmelen et al., 2014). In the current study, greater functional connectivity between the right amygdala and bilateral IFG, anterior cingulate cortex, and frontopolar cortex was associated with anxiety following threat exposure (Gold et al., 2015). However, compared with the MR group, the relative activation of the left lateral amygdala by IFG in the MD group was significantly decreased. That is, under chronic stress (maternal deprivation), IFG activation was suppressed. Hence, the brains of the monkeys in the MD group became less rational and more impulsive. A previous study reported that stress hormone levels were negatively related to the left IFG (Jahn et al., 2010), which is consistent with the results of this study. Early long‐term MD might be a kind of chronic stress state for MD monkeys (Feng et al., 2011), which leads to persistent inhibition of activity in the left IFG. As the MR monkeys had not been exposed to this kind of stressor, under the same conditions as the activated amygdala, the MD monkeys showed relatively lower activation in the left IFG than the MR monkeys. As a result, the functional coupling of the two structures in MD monkeys was decreased.

If anxious behaviors were affected by the corresponding neural connections, what factors in turn determine this network facet? The decrease in the coupling intensity of the left lateral amygdala (red cluster) and IFG in MD monkeys might be partly due to chronic stress (maternal deprivation; Gee et al., 2013). According to previous literature, cortisol can be an effective response to intrinsic chronic stress states. The cortisol level of MD monkeys was significantly higher than that of MR monkeys, definitely indicating the inner stressed state of the MD monkeys (Qin et al., 2015). Cortisol plays a biological function through its combination with the glucocorticoid receptor (Ishikawa et al., 2015; Moriceau et al., 2004, 2006; Reis et al., 2016), which is scattered universally in the brain (Tanaka et al., 2019), such as in the prefrontal lobe. For example, these combinations lead to the physiological secretion of neurotransmitters and the normal activity of synapses in the prefrontal lobe (Almada et al., 2015). However, under chronic stress, the cortisol level remained elevated (Chu et al., 2014). The secretion of neurotransmitters was disturbed, and synaptic transmission became abnormal (Di Cristo & Chattopadhyaya, 2020; Li et al., 2015). Therefore, the dysfunction of neural networks based on synaptic activity may be the mechanism behind the abnormal behaviors in MD monkeys in this study. In rats, on the other hand, low levels of stress hormones attenuated the affective response of the amygdala and thus prevented its hyperactivity, demonstrating a protective role in its connection to the frontal lobe (Moriceau et al., 2006). This may explain the high binding strength of the amygdala and IFG functions in MR monkeys, which might be partly ascribed to their lower cortisol levels than those of the MD monkeys.

This study reveals the important role of functional networks in abnormal behaviors in monkeys experiencing early adversity. Specifically, the mechanism of neural circuit alteration related to the abnormal change of emotional behaviors in MD monkeys was established. Hence, the normalization of the regions involved in the functional connection might reverse behavioral abnormality, which would provide a solid basis for effective intervention in humans’ early adversity.

## 6 CONCLUSION

This study suggests that early adversity‐induced anxious behaviors are associated with changes in the strength of the amygdala–prefrontal connection. The higher the amygdala–prefrontal connection strength, the less stereotyped behaviors exhibited by monkeys experiencing early adversity. Changing the strength of the amygdala–prefrontal connection may reverse the behavioral abnormalities of individuals who experience early adversity. It provides a solid foundation for effective intervention in humans’ early adversity.

## AUTHOR CONTRIBUTIONS

Each author has participated sufficiently to accept responsibility for the content of the manuscript. *Conceptualization*: Xiao‐Li Feng, Xin‐Tian Hu, and Tian‐Zi Jiang. *Data acquisition*: Xiao‐Feng Ren, Jie Zhang, Sheng‐Hai Wang, Yun Wang, and Zheng‐Fei Hu. *Analyzed and interpretation of data*: Jiao‐Jian Wang, Jing Wu, and Hui Zhou. *Writing—original draft*: Xiao‐Li Feng, Xiao‐Feng Ren, and Si‐Yu Li. Writing—review and editing: Xin‐Tian Hu and Tian‐Zi Jiang.

## CONFLICT OF INTEREST STATEMENT

The authors declare no conflicts of interest.

### PEER REVIEW

The peer review history for this article is available at https://publons.com/publon/10.1002/brb3.3027.

## Data Availability

The data that support the findings of this study are available from the corresponding author upon reasonable request.
